# High quality draft genome sequence of the slightly halophilic bacterium *Halomonas zhanjiangensis* type strain JSM 078169^T^ (DSM 21076^T^) from a sea urchin in southern China

**DOI:** 10.4056/sigs.5449586

**Published:** 2014-04-11

**Authors:** Yu Zhou, Rui Li, Xiao-Yang Gao, Alla Lapidus, James Han, Matthew Haynes, Elizabeth Lobos, Marcel Huntemann, Amrita Pati, Natalia N. Ivanova, Manfred Rohde, Konstantinos Mavromatis, Brian J. Tindall, Victor Markowitz, Tanja Woyke, Hans-Peter Klenk, Nikos C. Kyrpides, Wen-Jun Li

**Affiliations:** 1 Key Laboratory of Microbial Diversity in Southwest China, Ministry of Education and the Laboratory for Conservation and Utilization of Bio-Resources, Yunnan Institute of Microbiology, Yunnan University, Kunming, China; 2 State Key Laboratory Breeding Base for Zhejiang Sustainable Plant Pest Control; Institute of Quality and Standard for Agro-products, Zhejiang Academy of Agricultural Sciences, Hangzhou, Zhejiang, China; 3 Key Laboratory of Biogeography and Bioresource in Arid Land, Xinjiang Institute of Ecology and Geography, Chinese Academy of Sciences, Urumqi, China; 4 University of Chinese Academy of Sciences, Beijing, China; 5 Theodosius Dobzhansky Center for Genome Bionformatics, St. Petersburg State University, St. Petersburg, Russia; 6 Algorithmic Biology Lab, St. Petersburg Academic University, St. Petersburg, Russia; 7 DOE Joint Genome Institute, Walnut Creek, California, USA; 8 HZI – Helmholtz Centre for Infection Research, Braunschweig, Germany; 9 Leibniz-Institute DSMZ - German Collection of Microorganisms and Cell Cultures, Braunschweig, Germany; 10 Biological Data Management and Technology Center, Lawrence Berkeley National Laboratory, Berkeley, California, USA; 11 Department of Biological Sciences, King Abdulaziz University, Jeddah, Saudi Arabia

**Keywords:** strictly aerobic, motile Gram-negative, chemoorganotrophic, slightly halophilic, *Halomonadaceae*

## Abstract

*Halomonas zhanjiangensis* Chen *et al*. 2009 is a member of the genus *Halomonas*, family *Halomonadaceae*, class *Gammaproteobacteria*. Representatives of the genus *Halomonas* are a group of halophilic bacteria often isolated from salty environments. The type strain *H. zhanjiangensis* JSM 078169^T^ was isolated from a sea urchin (*Hemicentrotus pulcherrimus*) collected from the South China Sea. The genome of strain JSM 078169^T^ is the fourteenth sequenced genome in the genus *Halomonas* and the fifteenth in the family *Halomonadaceae*. The other thirteen genomes from the genus *Halomonas* are *H. halocynthiae*, *H. venusta*, *H. alkaliphila*, *H. lutea*, *H. anticariensis*, *H. jeotgali*, *H. titanicae*, *H. desiderata*, *H. smyrnensis*, *H. salifodinae*, *H. boliviensis*, *H. elongata* and *H stevensii*. Here, we describe the features of strain JSM 078169^T^, together with the complete genome sequence and annotation from a culture of DSM 21076^T^. The 4,060,520 bp long draft genome consists of 17 scaffolds with the 3,659 protein-coding and 80 RNA genes and is a part of *Genomic Encyclopedia of Type Strains*, Phase I: the one thousand microbial genomes (KMG) project.

## Introduction

Strain JSM 078169^T^ (= DSM 21076 = KCTC 22279 = CCTCC AB 208031) is the type strain of the species *Halomonas zhanjiangensis* [1], one out of 84 species with a validly published name in the genus *Halomonas* [2], family *Halomonadaceae* [3]. The family *Halomonadaceae* currently comprises thirteen genera (*Aidingimonas*, *Carnimonas*, *Chromohalobacter*, *Cobetia*, *Halomonas*, *Halotalea*, *Halovibrio*, *Kushneria*, *Marinospirillum*, *Modicisalibacter*, *Candidtus* Portiera, *Salinicola* and *Zymobacter*) with *Halomonas* being the largest genus in this family [3-6]. Members of the genus *Halomonas* have been isolated from various saline environments and showed halophilic characteristics [7-11]. Strain JSM 078169^T^ was originally isolated from a sea urchin (*Hemicentrotus pulcherrimus*) that was collected from the South China Sea. The genus name was derived from the Greek words ‘halos’ meaning ‘salt’ and ‘monas’ meaning ‘monad’, yielding the Neo-Latin word ‘halomonas’ [2]; the species epithet was derived from Latin word ‘zhanjiangensis’, of Zhanjiang, a city in China near where the sample was collected [1]. Strain JSM 078169^T^ was found to assimilate several mono- and disaccharides and to produce numerous acid and alkaline phosphatases, leucine arylamidase, naphthol-ASBI-phosphohydrolase and valine arylamidase [1]. There are no PubMed records that document the use of these strain for any biotechnological studies; only comparative analyses performed for the description of later members of the genus *Halomonas* are recorded. However, the NamesforLife [12] database reports at least 70 patents in which *Halomonas* ssp. are referenced. Here we present a summary classification and a set of feature for *H. zhanjiangensis* JSM 078169^T^, together with the description of the genomic sequencing and annotation of DSM 21076.

## Classification and features

### 16S rRNA analysis

The original assembly of the genome did not contain longer stretches of 16S rRNA copies. Therefore, a 1,413 bp long fragment of the 16S rRNA gene was later patched into the genome sequence assembly. This almost full length version of the 16S rRNA sequence was compared using NCBI BLAST [13,14] under default settings (e.g., considering only the high-scoring segment pairs (HSPs) from the best 250 hits) with the most recent version of the Greengenes database [15] and the relative frequencies of taxa and unidentified clones (or strains) were calculated by BLAST scores. The most frequently occurring genus was *Halomonas* (74.8%), and the unidentified clones or isolates represented 25.5% for the total BLAST results. Except for sequences of representatives of the genus *Halomonas*, no sequences from other genera were observed in the BLAST search. The highest degree of sequence similarity was reported with *H. alkantarctica* str. CRSS.

**Figure 1:**
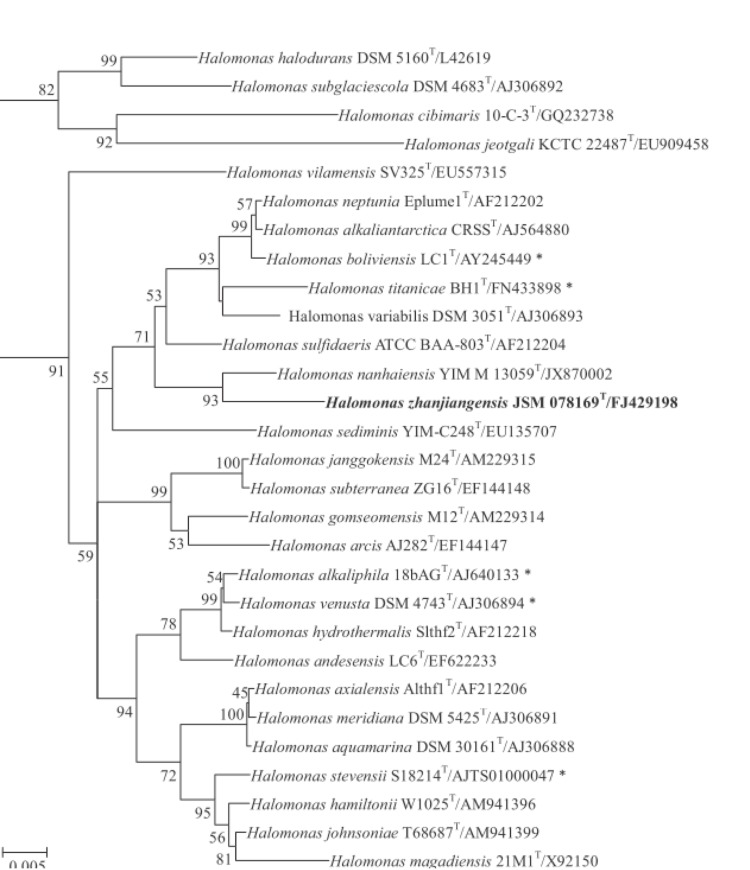
Phylogenetic tree highlighting the position of *H. zhanjiangensis* relative to the closest related type strains of the other species within the family *Halomonadaceae*. All the 16S rRNA gene sequences of the type strains within the genus *Halomonas* were included and combined with the representative 16S rRNA gene sequences of the type species in other genera, according to the most recent release of the EzTaxon database. The tree was inferred from 1,381 aligned characters [16] under the neighbor-joining (NJ) [17], and maximum-likelihood (ML) [18] method with 1,000 randomly selected bootstrap replicates using MEGA version 5.2 [19]. The branches are scaled in terms of the expected number of substitutions per site. Numbers adjacent to the branches are support values from 1,000 NJ bootstrap (left) and from 1,000 ML bootstrap (right) replicates [20] if larger than 60%. Lineages with type strain genome sequencing projects registered in GOLD [21] are labeled with one asterisk, those also listed as ‘Complete and Published’ with two asterisks [22].

Figure 1 shows the phylogenetic neighborhood of *H. zhanjiangensis* JSM 078169^T^ in a tree based on 16S rRNA genes. The 1,413 bp long sequence fragment of the 16S rRNA gene differs by three nucleotides from the previously published 16S rRNA sequence (FJ429198). The tree provided a precise insight into the nomenclature and classification of members of the genus *Halomonas*. The phylogenetic analysis showed that strain *H. zhanjiangensis* JSM 078169^T^ was most closely related to *H. nanhaiensis* YIM M 13059^T^ with 98.3% sequence similarity.

### Morphology and physiology

**Figure 2:**
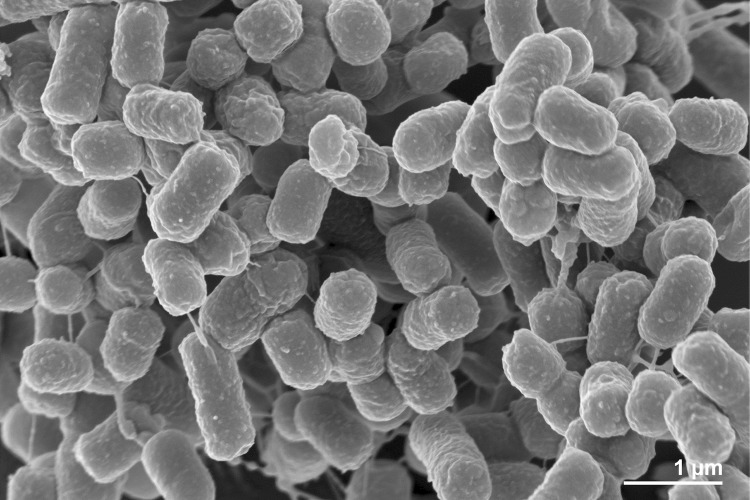
Scanning electron micrograph of *H. zhanjiangensis* JSM 078169^T^

*H. zhanjiangensis* JSM 078169^T^ is a Gram-negative-staining, non-sporulating, strictly aerobic (Table 1), catalase-positive, oxidase-negative and slightly halophilic bacterium that reduces nitrate [1]. Cells of JSM 078169^T^ are short rods (0.4-0.7 μm × 0.6-1.0 μm) and motile with peritrichous flagella (not visible in Figure 2). Colonies are yellow-pigmented, flat and non-translucent with glistening surfaces and circular/slightly irregular margins, 2-3 mm in diameter after incubation on Marine Agar (MA) at 28 ºC for 3-5 days. No diffusible pigments are produced. Growth occurs at 4-40 ºC with an optimum growth at 25-30 ºC, at pH range of 6.0-10.5 with an optimum pH of 7.5. The salinity range suitable for growth was 1.0-20.0% (w/v) total salts with an optimum between 3.0-5.0% (w/v) total salts. No growth occurs in the absence of NaCl or with NaCl as the sole salt. Strain JSM 078169^T^ grows on Marine Agar and the medium contained the following: 5.0 g peptone, 1.0 g yeast extract, 0.1 g ferric citrate, 19.45 g NaCl, 8.8 g MgCl_2_, 3.24 g Na_2_SO_4_, 1.8 g CaCl_2_, 0.55 g KCl, 0.16 g NaHCO_3_, 0.08 g KBr, 0.034 g SrCl_2_, 0.022 g H_3_BO_3_, 0.004 g sodium silicate, 0.0024 g sodium fluoride, 0.0016 g ammonium nitrate, 0008 g disodium phosphate and 15 g agar.

**Table 1:** Classification and general features of *H. zhanjiangensis* JSM 078169^T^ according the MIGS recommendations [23], (published by the Genomic Standards Consortium [24]), List of Prokaryotic names with Standing in Nomenclature [25] and the Names for Life database [12].

**MIGS ID**	**Property**	**Term**	**Evidence code**
MIGS-2	Domain	*Bacteria*	TAS [26]
	Phylum	*Proteobacteria*	TAS [27]
	Class	*Gammaproteobacteria*	TAS [28-30]
	Order	*Oceanospirillales*	TAS [29,31]
MIGS-3	Family	*Halomonadaceae*	TAS [3,32-35]
	Genus	*Halomonas*	TAS [2,36]
	Species	*Halomonas zhanjiangensis*	TAS [1]
	Type strain	JSM 078169	TAS [1]
MIGS-37.1	Cell shape	rod-shaped	TAS [1]
MIGS-37.2	Motility	motile	TAS [1]
MIGS-37.3	Sporulation	non-sporulating	TAS [1]
MIGS-37.12	Temperature range	4-40°C	TAS [1]
	Optimum temperature	25-30°C	TAS [1]
	Salinity	1-20% NaCl (w/v), optimum 3-5%	TAS [1]
	pH	6.0-10.5	TAS [1]
MIGS-37.5	Cell diameter	0.7-1.4 μm	TAS [1]
MIGS-37.6	Cell length	1.5-2.5 μm	TAS [1]
MIGS-37.9	Cell arrangement	singles, pairs	TAS [1]
MIGS-37.11	Energy metabolism	chemoorganotrophic	TAS [1]
MIGS-6	Habitat	aquatic, marine host	TAS [1]
MIGS-22	Oxygen requirement	aerobe	TAS [1]
MIGS-15	Biotic relationship	not reported	TAS [1]
MIGS-16	Host name	*Hemicentrotus pulcherrimus* (sea urchin)	TAS [1]
MIGS-4.5	Isolation site		TAS [1]
MIGS-4	Geographic location	Naozhou island, South China Sea near Zhanjiang	TAS [1]
MIGS-4.1	Latitude	20.90	TAS [1]
MIGS-4.2	Longitude	110.59	TAS [1]
MIGS-4.3	Altitude	not reported	
MIGS-4.4	Depth	not reported	

Evidence codes-TAS: Traceable Author Statement (i.e., a direct report exists in the literature); NAS: Non-traceable Author Statement (i.e., not directly observed for the living, isolated sample, but based on a generally accepted property for the species, or anecdotal evidence). Evidence codes are from the Gene Ontology project [37].

### Chemotaxonomy

The predominant respiratory quinone is Q-9 which is consistent to the other members of the genus *Halomonas* [1]. The predominant fatty acids are C_18:1_ *_ω_*_7_*_c_* (48.9%), C_16:0_ (17.0%) and C_12:0 3-OH_ (10.7%). The profile of major fatty acids is also similar to the other representatives of the genus *Halomonas* [38-41].

## Genome sequencing and annotation

### Genome project history

This organism was selected for sequencing on the basis of its phylogenetic position [42,43]. Sequencing strain JSM 078169^T^ is part of *Genomic Encyclopedia of Type Strains*, Phase I: the one thousand microbial genomes (KMG) project [44], a follow-up of the GEBA project [45], which aims in increasing the sequencing coverage of key reference microbial genomes. The genome project is deposited in the Genomes OnLine Database [21] and the permanent draft genome sequence is deposited in GenBank. Sequencing, finishing and annotation were performed by the DOE Joint Genome Institute (JGI) using state of the art sequencing technology [46]. A summary of the project information is shown in Table 2.

**Table 2:** Genome sequencing project information

**MIGS ID**	**Property**	**Term**
MIGS-31.1	Sequencing quality	Level 2: High-Quality Draft
MIGS-28.1	Libraries used	One Illumina standard shotgun library
MIGS-29	Sequencing method	Illumina HiSeq 2000,
MIGS-30 MIGS-32	Assembly method Gene calling method INSDC ID Genbank date of release GOLD ID	Velvet v. 1.1.04; ALLPATHS v. r41043 Prodigal ARIT00000000 December 12, 2013 Gi11554
MIGS-1.1	NCBI project ID	178047
MIGS-1.2	Straininfo ID	845770
	Database: IMG	2517572236
MIGS-13	Source material identifier	DSM 21076
MIGS-38.2	Project relevance	Tree of Life, GEBA-KMG

### Growth conditions and DNA isolation

*H. zhanjiangensis* JSM 078169^T^, DSM 21076, was grown in DSMZ medium 1510 (modified medium 514 for *Halomonas sp.*) [47] at 28 ºC. DNA was isolated from 0.5-1.0 g of cell paste using MasterPure Gram-positive DNA purification kit (Epicentre MGP04100) following the standard protocol as recommended by the manufacturer with modification st/DL for cell lysis as described by Wu *et al.* [45]. DNA is available through the DNA Bank Network [48].

### Genome sequencing and assembly

The draft genome sequence was generated using the Illumina technology [49]. An Illumina Standard shotgun library was constructed and sequenced using the Illumina HiSeq 2000 platform which generated 15,593,002 reads totaling 2,339.0 Mbp. All general aspects of library construction and sequencing performed at the JGI can be found at [50]. All raw Illumina sequence data was passed through DUK, a filtering program developed at JGI, which removes known Illumina sequencing and library preparation artifacts [51]. Following steps were then performed for assembly: (1) filtered Illumina reads were assembled using Velvet [52], (2) 1–3 kbp simulated paired end reads were created from Velvet contigs using wgsim [53], (3) Illumina reads were assembled with simulated read pairs using Allpaths–LG [54]. Parameters for assembly steps were: 1) Velvet (velveth: 63 –shortPaired and velvetg: –very clean yes –export-Filtered yes –min contig lgth 500 –scaffolding no –cov cutoff 10) 2) wgsim (–e 0 –1 100 –2 100 –r 0 –R 0 –X 0) 3) Allpaths–LG (PrepareAllpathsInputs: PHRED 64=1 PLOIDY=1 FRAG COVERAGE=125 JUMP COVERAGE=25 LONG JUMP COV=50, RunAllpathsLG: THREADS=8 RUN=std shredpairs TARGETS=standard VAPI WARN ONLY=True OVERWRITE=True). The final draft assembly contained 18 contigs in 17 scaffolds. The total size of the genome is 4.1 Mbp and the final assembly is based on 501.3 Mbp of Illumina data, which provides an average 123.5 × coverage of the genome.

### Genome annotation

Genes were identified using Prodigal [55] as part of the DOE-JGI genome annotation pipeline [56], following by a round of manual curation using the JGI GenePRIMP pipeline [57]. The predicted CDSs were translated and used to search the National Center for Biotechnology Information (NCBI) non-redundant database, UniProt, TIGR-Fam, Pfam, PRIAM, KEGG, COG, and InterPro database. These data sources were combined to assert a product description for each predicted protein. Additional gene prediction analysis and functional annotation was performed within the Integrated Microbial Genomes-Expert Review (IMG-ER) platform [58].

### Genome properties

**Figure 3:**
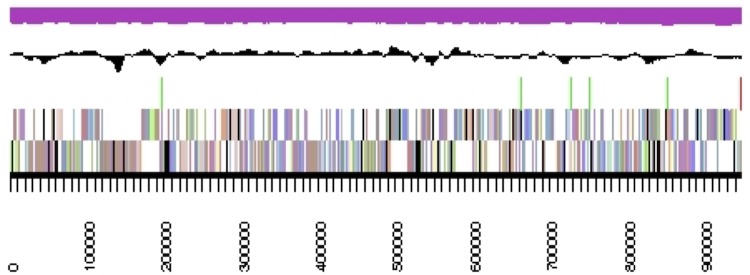
The graphical map of the largest scaffold of the genome. From bottom to the top: Genes on forward strand (color by COG categories), Genes on reverse strand (color by COG categories), RNA genes (tRNA green, rRNA red, other RNAs black), GC content, GC skew (purple/olive).

The assembly of the draft genome sequence consists of 17 scaffolds amounting to 4,060,520 bp, and the G+C content is 54.5% (Table 3 and Figure 3). Of the 3,739 genes predicted, 3,659 were protein-coding genes, and 80 RNAs. The majority of the protein-coding genes (87.1%) were assigned a putative function while the remaining ones were annotated as hypothetical proteins. The distribution of genes into COGs functional categories is presented in Table 4 and Figure 3.

**Table 3:** Genome statistics

**Attribute**	Value	% of total
Genome size (bp)	4,060,520	100.00%
DNA coding region (bp)	3,661,597	90.18%
DNA G+C content (bp)	2,212,212	54.48%
Number of scaffolds	17	
Extrachromosomal elements	unknown	
Total genes	3,739	100.00%
RNA genes	80	2.14%
rRNA operons	unknown	
tRNA genes	56	1.50%
Protein-coding genes	3,659	97.86%
Pseudo genes	0	0.00%
Genes with function prediction (proteins)	3,256	87.08%
Genes in paralog clusters	2,856	76.38%
Genes assigned to COGs	3,175	84.92%
Genes assigned Pfam domains	3,303	88.34%
Genes with signal peptides	313	8.37%
Genes with transmembrane helices	941	25.17%
CRISPR repeats	1	

**Table 4:** Number of genes associated with the general COG functional categories

**Code**	**Value**	**% age**	**Description**
J	180	5.03	Translation, ribosomal structure and biogenesis
A	1	0.03	RNA processing and modification
K	251	7.02	Transcription
L	143	4.00	Replication, recombination and repair
B	6	0.17	Chromatin structure and dynamics
D	38	1.06	Cell cycle control, cell division, chromosome partitioning
Y	0	0.00	Nuclear structure
V	36	1.01	Defense mechanisms
T	168	4.70	Signal transduction mechanisms
M	206	5.76	Cell wall/membrane/envelope biogenesis
N	93	2.60	Cell motility
Z	0	0.00	Cytoskeleton
W	0	0.00	Extracellular structures
U	80	2.24	Intracellular trafficking, secretion, and vesicular transport
O	137	3.83	Posttranslational modification, protein turnover, chaperones
C	211	5.90	Energy production and conversion
G	267	7.47	Carbohydrate transport and metabolism
E	342	9.56	Amino acid transport and metabolism
F	77	2.15	Nucleotide transport and metabolism
H	173	4.84	Coenzyme transport and metabolism
I	145	4.05	Lipid transport and metabolism
P	217	6.07	Inorganic ion transport and metabolism
Q	95	2.66	Secondary metabolites biosynthesis, transport and catabolism
R	404	11.30	General function prediction only
S	306	8.56	Function unknown
-	563	15.06	Not in COGs

## Insights into the genome sequence

One complete genome sequence from a type strain of the family *Halomonas* - *H. elongata* [22] is available in GenBank, and four other permanent draft genomes of *H. anticariensis*, *H. lutea*, *H. jeotgali* and *H. halocynthiae* are available from IMG. The genome size of *H. zhanjiangensis* is smaller than those of *H. elongata*, *H. lutea* and *H. anticariensis* (4.06-5.02 Mbp), but much larger than those of *H. jeotgali* and *H. halocynthiae* (2.85-2.88 Mbp). Using the genome-to-genome distance calculator [59-61] version 2.0 revealed that all digital DNA-DNA hybridization (DDH) values are much lower than 70% using the program NCBI-BLAST, which demonstrated that *H. zhanjiangensis* is distinct from *H. elongata*, *H. anticariensis*, *H. lutea*, *H. jeotgali* and *H. halocynthiae* at the species level. Distance is 0.1845 between the type strain genomes of *H. zhanjiangensis* and *H. elongata*, which corresponds to a DDH value of 13.00 ± 2.99%. The distances of *H. zhanjiangensis* from *H. anticariensis*, *H. lutea*, *H. jeotgali* and *H. halocynthiae* are 0.1842, 0.1837, 0.1835 and 01849, which correspond to DDH values of 20.30 ±2.41%, 20.30 ±2.41%, 20.40 ±2.41% and 20.20 ±2.41%, respectively.

A major feature of the previously sequenced genomes from this family is the presence of large numbers of proteins for the TRAP-type C4-dicarboxylate transport systems. A total of 267 genes in the genome of *H. zhanjiangensis* encode proteins for carbohydrate transport and metabolism, 68 genes are related to TRAP-type C4-dicarboxylate transport systems and encoded 22 large permease proteins, 24 periplasmic proteins and 22 small permease proteins. Genomic analysis of *H. elongata*, *H. anticariensis*, *H. lutea*, *H. jeotgali* and *H. halocynthiae* showed that they encode 58, 65, 61, 7 and 32 proteins related to TRAP-type C4-dicarboxylate transport system respectively. Proteins for TRAP-type C4-dicarboxylate transport systems constitute 1.86% as the total protein-coding sequences of the *H. zhanjiangensis* genome. In the genomes of *H. elongata*, *H. anticariensis*, *H. lutea*, *H. jeotgali* and *H. halocynthiae*, TRAP-type C4-dicarboxylate transport system related proteins are accounted for 1.67%, 1.37%, 1.42%, 0.27% and 1.18% of the total protein-coding genes respectively. Therefore, *H. zhanjiangensis* has the highest percentage of TRAP-type C4-dicarboxylate transport system related encoding proteins in this group of bacteria to date.

Of the signal transduction mechanisms, Methyl-accepting Chemotaxis Proteins (MCPs) are transmembrane sensor proteins of bacteria. The MCPs allow bacteria to detect concentrations of molecules in the extracellular matrix so that they may smoothly swim or tumble accordingly [62,63]. Various environmental conditions give rise to diversity in bacterial signaling receptors, and consequently there are many genes encoding MCPs [64]. A number of MCPs (23) are present in *H. zhanjiangensis*, while *H. elongata*, *H. anticariensis*, *H. lutea*, and *H. jeotgali* have only 4, 21, 16, and 17 MCPs, respectively. MCPs are not found in the genome of *H. halocynthiae*. *H. zhanjiangensis* has the largest numbers of MCPs in this family. The analysis of bacterial genomes reveals that the family *Halomonadaceae* differs enormously in the number of MCPs from *E. coli*, and the number of MCPs in *Halomonadaceae* is about two times than that of *E. coli* strains.
